# Jejunal gastrointestinal stromal tumor (GIST) with profound anemia

**DOI:** 10.1093/jscr/rjae497

**Published:** 2024-08-20

**Authors:** Amelia T Huynh, Ann Rust

**Affiliations:** Department of Surgery, Good Shepherd Health Care System, 610 NW 11th St., Hermiston, OR 97838, United States; School of Osteopathic Medicine, Pacific Northwest University of Health Sciences, Butler-Haney Hall, 200 University Pkwy, Yakima, WA 98901, United States; Department of Surgery, Good Shepherd Health Care System, 610 NW 11th St., Hermiston, OR 97838, United States; School of Osteopathic Medicine, Pacific Northwest University of Health Sciences, Butler-Haney Hall, 200 University Pkwy, Yakima, WA 98901, United States

**Keywords:** gastrointestinal stromal tumor, jejunum, small bowel resection, primary anastomosis, laparotomy, bleeding, computed tomography

## Abstract

Gastrointestinal stromal tumors (GISTs) are uncommon tumors typically found in the stomach, with an even rarer appearance in the jejunum. These tumors are often discovered incidentally, given their nonspecific presentation. We present a case of chronic iron deficiency anemia in a patient with symptomatic GIST involving the proximal jejunum requiring robot-assisted resection with primary anastomosis. Pathological examination of the excised GIST revealed positive immunoreactivity for cKIT, DOG1, and CD37. This case highlights the importance of considering GIST as a differential diagnosis for chronic anemia and emphasizes the critical role of CT imaging in its detection and management.

## Introduction

Gastrointestinal stromal tumors (GISTs) represent a rare subset of mesenchymal tumors, comprising merely 1% of all gastrointestinal neoplasms, and predominantly manifest within the stomach (60%) and small intestine (20%–30%) [1, 2]. Incidentally detected typically between the ages of 60 and 65, these tumors exhibit a nuanced predilection toward males, who notably present with larger tumor dimensions and a heightened propensity for metastasis [3]. Treatment strategies for jejunal GISTs consider parameters, such as tumor size, mitotic rate, and location, with radical resection as the therapeutic cornerstone. GISTs are distinguished by cluster differentiation (CD)117 and CD34 positivity, guiding diagnosis and therapy, with imatinib mesylate resulting in improved patient outcomes [2, 4, 5].

The patient provided written consent for the report of his clinical care details and imaging studies.

## Case report

A 67-year-old male with a past medical history of hypertension, heartburn, peptic ulcer disease, and long-standing iron deficiency anemia (IDA) presented to the emergency department with a 5-day history of melena with associated dizziness, headache, and asthenia. Previous surgical history included cholecystectomy. On initial presentation, he was afebrile with positive findings for orthostatic hypotension, including blood pressures of 140/76 mmHg sitting and 99/60 mmHg standing. Initial laboratory findings were significant for hemoglobin (Hgb) of 4.4 g/dl, hematocrit of 12.6%, hypokalemia at 2.7 mEq/L, hypocalcemia at 7.6 mg/dl, white blood cell (WBC) of 25.2 × 103/mcl, troponin 0.66 ng/ml, myoglobin 227 mcg/L, and CK-MB 5.4%. The patient received two units of packed red blood cells, and his Hgb improved to Hgb 7.2 g/dl. Urgent esophagoduodenoscopy unveiled a 4 cm hiatal hernia with normal gastric and duodenal biopsies, suggesting upper gastrointestinal bleeding stemming from the small bowel. Subsequently, he consented to admission for further workup.

**Figure 1:**
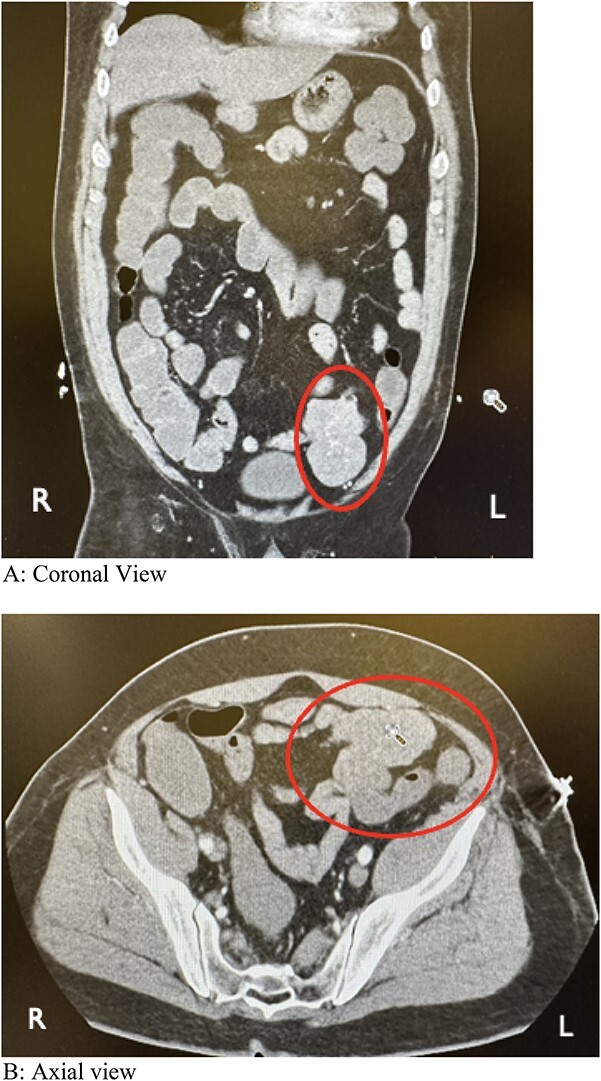
Computed tomography angiography showing a lobulated soft tissue mass associated with distal small bowel loops in the left lower quadrant. (A) Coronal view. (B) Axial view.

During colonoscopy, the mucosa of the entire colon appeared unremarkable. However, black tarry stool was detected, extending from the transverse colon to ~20–25 cm into the terminal ileum, which was successfully intubated. Despite a thorough examination, no bleeding source or additional abnormalities were identified. Subsequent computed tomography angiography (CTA) of the abdomen and pelvis revealed a lobulated soft tissue mass adjacent to the distal small bowel loop, measuring 5.7 × 3.5 × 7.4 cm, concerning for small bowel tumor versus mesenteric tumor in the left lower quadrant (LLQ) (Fig. 1A and B). Following discussion of the CT results, the patient, who had been initially reluctant to provide his medical history, described experiencing a slowly progressive sensation of dull ache and bulge in the LLQ since his teenage years, accompanied by intermittent episodes of abdominal cramping and pain, along with changes in stool patterns. Despite experiencing these symptoms over several decades, investigations were conducted intermittently without imaging, with the symptoms being attributed to functional causes without identifying an underlying cause.

The patient consented to robotic-assisted exploratory laparotomy, revealing a smooth, highly vascularized mass involving the proximal jejunum. No metastatic deposits were found upon thorough abdominal inspection, including peritoneal walls, mesentery, small bowel, colon, and liver. The considerable weight of the mass caused the jejunum to descend to the LLQ. The small bowel was lifted with a mesenteric window created proximal to the mass. The bowel was divided with a blue load stapler and the mesentery was separated using a vessel sealer. The adhesion-free mass was resected along with the remaining mesentery and placed in an endopouch. Bowel edge perfusion was evaluated with Indocyanine Green, followed by preparation for anastomosis with a blue load stapler in a side-to-side antiperistaltic fashion. The anastomosis was carefully examined for closure, twists, stool spillage, and vascular integrity before removal. The patient’s postoperative course was uneventful, and he was subsequently discharged on postoperative Day 3. At his 1-week and 1-month follow-up, he had no complaints of pain and was able to ambulate without issues.

Pathologic assessment revealed a firm, irregularly shaped lobulated mass measuring 9.0 × 5.5 × 5.5 cm, identified as a spindle cell type GIST. Immunostains were positive for cKIT (CD117), GIST-1 (DOG1) antigens, and CD37. Microscopic evaluation classified it as Grade 1, with a moderate risk, showing one mitosis per 5 mm^2^. Surgical margins were clear of the tumor. Despite recommendations for adjuvant Imatinib therapy, the patient declined, citing confidence in managing the tumor he had for over 50 years. Instead, he opted for annual CT imaging surveillance given his resolved symptoms.

## Discussion

The absence of imaging in the patient’s medical history led to missed opportunities for early intervention. Despite presenting with symptoms suggestive of gastrointestinal bleeding, solely characterizing his condition as IDA without further investigation is inadequate, but there is a notable paucity of comprehensive clinical guidelines for managing IDA with GI bleed. While GISTs can be asymptomatic, they often present with GI bleeding, abdominal discomfort, mass, mechanical obstruction, and hemorrhage [6, 7]. Diagnosing jejunal GISTs is challenging due to vague symptoms and difficulties in localization with standard endoscopic methods, leading to delayed diagnosis. Contrast-enhanced CT of the abdomen and pelvis emerges as the preferred diagnostic tool, surpassing magnetic resonance imaging (MRI) in detailing the small intestine’s anatomy, with 95% accuracy for lesions over 2 cm [2, 5]. Recent studies highlight CT’s effectiveness in detecting hypodense hypervascular masses in the jejunum, even with normal endoscopic results [7, 8]. Comparatively, double-balloon endoscopy (DBE) offers similar sensitivity but is invasive and less accessible [9]. Thus, CT remains the primary diagnostic modality for jejunal GISTs, offering important insights even when endoscopy fails to detect abnormalities.
